# Screening of Virulence-Related Transcriptional Regulators in *Streptococcus suis*

**DOI:** 10.3390/genes11090972

**Published:** 2020-08-21

**Authors:** Liang Liu, Qiang Zhang, Zhongmin Xu, Bo Chen, Anding Zhang, Xiaomei Sun, Meilin Jin

**Affiliations:** 1 Unit of Animal Infectious Diseases, National Key Laboratory of Agricultural Microbiology, College of Veterinary Medicine, Huazhong Agricultural University, Wuhan 430070, China; robin2371@163.com (L.L.); 05yisan@163.com (Q.Z.); xzmjack@126.com (Z.X.); hzauchen@gmail.com (B.C.); andye8019@mail.hzau.edu.cn (A.Z.); Sunxm320@126.com (X.S.); 2 Key Laboratory of Preventive Veterinary Medicine in Hubei Province, The Cooperative Innovation Center for Sustainable Pig Production, Wuhan 430070, China; 3 Key Laboratory of Development of Veterinary Diagnostic Products, Ministry of Agriculture, Wuhan 430070, China

**Keywords:** *Streptococcus suis*, transcriptional regulator, in vivo, virulence, mutant

## Abstract

*Streptococcus suis* (*S.suis*) is an important zoonotic pathogen that causes many severe diseases in pigs and humans. Virulence-related transcriptional regulators have been widely reported in pathogenic microorganisms, but only a few have been identified in *S.suis*. Our aim was to screen virulence-related transcriptional regulators in *S.suis*. A total of 89 such genes were predicted in the *S.suis* genome, of which 22 were up-regulated and 18 were down-regulated during *S.suis* infection in mice. To evaluate the roles of these differentially expressed factors in *S.suis* virulence, deletion mutants were constructed, and 10 mutants were successfully obtained. Among these genes, the deletion of *comR*, *sitR*, or *sxvR* caused significantly decreased virulence in mice, compared to that with the wild-type strain. Moreover, the survival of Δ*comR*, Δ*sitR*, and Δ*sxvR* mutant strains in blood was significantly reduced both in vitro and in vivo. Furthermore, their pro-inflammatory abilities were also obviously decreased in vivo. The regulatory mechanisms of *comR*, *sitR*, and *sxvR* were then analyzed by whole transcriptome RNA sequencing (RNA-Seq). Results indicated that the absence of *comR* induced the down-regulation of 17 virulence factors or virulence-related factors, including genes involved in the synthesis of capsules, oxidative stress tolerance, immune evasion, and cell division. Furthermore, three and two virulence factors or virulence-related factors were down-regulated upon deletion of *sitR* and *sxvR*, respectively. Thus, this study reports the discovery of three virulence-associated transcriptional regulatory factors in *S.suis*. These factors could ultimately be targeted to control infection caused by these bacteria.

## 1. Introduction

*Streptococcus suis (S.suis)* is an important pathogen in pigs which can cause infection in pigs and people who contact with pigs and their by-products [1]. This pathogen, which can cause arthritis, meningitis, endocarditis, and many other pathologies, is responsible for severe economic losses worldwide in the swine industry and poses a serious threat to human health [2]. Since the first reported human case in Denmark, similar cases have been reported worldwide [3]. More than 1600 human cases of *S.suis* infections reported worldwide between 2002 and 2013, mainly in Asia (90.2%), and second in Europe (8.5%) [4]. Among the 29 described serotypes based on capsular antigens, serotype 2 (SS2) is most frequently isolated from diseased pigs in most countries [5]. Furthermore, two deadly human *S.suis* outbreaks, specifically in 1998 and 2005 have occurred in China [6]. Accordingly, SS2 has evolved into a serious pathogen, especially in patients with the streptococcal toxic shock-like syndrome; this suggests the recent emergence of new highly toxic bacterial variants in Asia [7].

The virulence of bacteria has been described as the ability to invade and replicate in the host and to evade the host’s immune system [8]. Furthermore, this process mainly includes adhesion, invasion, immune evasion, and inflammatory injury. Almost all reported virulence factors of *S.suis* are involved in these processes. For example, suilysin (SLY) functions to create pores in target cell membranes, facilitating bacterial colonization and further invasion into the host circulation [9,10]. Moreover, capsular polysaccharide (CPS) can protect *S.suis* from neutrophil and monocyte/macrophage-mediated phagocytosis and cytotoxicity [6]. In addition, the DNase *ssnA* contributes to the degradation of neutrophil extracellular traps (NETs) and the evasion of NET-mediated antimicrobial activity [11].

More than 100 virulence-related factors have been reported in SS2, encompassing almost all stages of infection [4]. However, the pathogenesis of SS2 still cannot be thoroughly explained, suggesting that the continued search for virulence-related genes in *S.suis* still might not result in a comprehensive view of its pathogenic mechanisms. Furthermore, it is well known that the pathogenicity of SS2 is a complex process involving a large number of virulence-related genes, and research on the regulatory mechanisms of such genes is expected to become a new direction for studying the associated pathogenic mechanisms.

Although the presence of some proposed virulence factors does not necessarily define specific *S.suis* strains as virulent, several cell-associated or secreted factors are clearly important for the pathogenesis of this microbe [12]. However, these factors do not correlate with virulence at all stages of the disease, and some factors even have completely opposing functions at different stages of infection. For example, CPS is considered an important virulence factor [13]. It can help *S.suis* evade macrophage-and neutrophil-mediated phagocytosis for survival in the host [14]. However, some experiments showed that *S.suis* downregulates the expression of the CPS in the early stages of infection to better adhere to the host surface [6,15]. In addition, some virulence-related factors, namely MRP, EF, SLY, GAPDH, and FBPS were found to be up-regulated when the bacteria were isolated from several organs of the body, and there were expression differences among organs [16]. These facts indicate that the virulence factor regulatory system plays an important role in infection and pathogenesis [17]. Furthermore, microorganisms encounter different microenvironments during host infection and respond to stimuli by differentially regulating the production of proteins necessary for a particular process [18]. Accordingly, transcriptional regulators play an important role in the response to environmental signals by modulating the expression of related genes [19].

Several transcriptional regulators which include transcription factors, two-component signal transduction (TCS) systems and orphan regulators have been reported to be associated with the virulence of *S.suis* [4]. For example, catabolite control protein A (CcpA) significantly affects capsule synthesis and the virulence properties of *S.suis*. Moreover, the orphan response regulator CovR is a negative global modulator of virulence in *S.suis*. In addition, Rgg in SS2 is a global transcriptional regulator that plays an important role in promoting bacterial survival during pathogen–host interactions [1]. However, how these factors affect virulence (and the extent of their regulatory effects) has not yet been elucidated [6].

To uncover the regulatory roles of transcription regulators that mediate the virulence of *S.suis* and to map the associated networks of such genes, additional virulence factors especially virulence-related transcriptional regulators need to be discovered. Here, we performed a whole-genome screen of *S.suis* to uncover novel virulence-related transcriptional regulators; further, we assessed virulence by in vivo induction, mutant construction, and animal experiments. We also analyzed the regulatory mechanisms of these identified factors by comparing transcriptional profiles between hypovirulent deletion mutants and the wild-type strain.

## 2. Materials and Methods

### 2.1. Bacterial Strains, Plasmids, and Growth Conditions

The bacterial strains and plasmids used in this study are listed in Table S1. SS2 strains were grown in tryptic soy broth medium or plated on tryptic soy agar (Difco, Detroit, MI, USA) containing 10% newborn bovine serum (Sijiqing Biological Engineering Materials Co. Ltd., Hangzhou, China). *Escherichia coli (E. coli)* DH5a was cultured in Luria–Bertani liquid medium or plated on Luria–Bertani agar. If required, spectinomycin was added to the plate or broth at the following concentrations: 100 μg/mL for *S.suis* serotype 2, 50 μg/mL for *E. coli* strain DH5a.

### 2.2. In Vivo-Induced Experiments

The in vivo-induced experiments were performed as previously described with some modifications [20]. Ten specific pathogen-free C57BL/6 mice were intraperitoneally infected with 5 × 10^8^ colony-forming unit (CFU) of the SC-19 (WT) strain. The mice were then sacrificed and dissected after 6 h. The spleens were harvested and immediately frozen in liquid nitrogen and the organs were stored at −80 °C. Prior to RNA isolation, the organs were thawed on ice and homogenized in 15 mL of an ice-cold solution composed of 0.2 M sucrose/0.01% SDS and 2μL Recombinant RNase Inhibitor (Takara, Shiga, Japan). The homogenate was gently centrifuged for 20 min at 300× *g* and filtered to remove large tissue debris. The tissue suspension was then centrifuged for 20 min at 4000 rpm to pellet the bacteria. Centrifugations were performed at 4 °C. After the removal of the supernatant, the pellet was resuspended and washed with PBS. As a control, 5 mL of in vitro WT strains were collected when the OD was 0.6, representing mid-stationary phase. Subsequently, 1 mL TRIzol^®^ reagent (Invitrogen, Paisley, UK) and 0.5 g of glass beads (0.2–0.3 mm in diameter) were added. The homogenate was proceeded with 4500 rpm for 20 s five times. The mixture was then transferred to a fresh tube and centrifuged at 12,000 rpm for 2 min at 4 °C. The supernatant was then collected, and bacterial RNA extraction was performed as previously described [21]. Quality of RNA was assessed by determining the OD260/280 ratio. Subsequently, complementary DNA was synthesized from 500 ng of total RNA using AMV reverse transcriptase (Takara), as previously described [22]. qRT-PCR was performed using ViiA™ 7 Software (Applied Biosystems, Waltham, MA, USA,) with a SYBR Green PCR Kit (Roche, Basel, Switzerland,). All primers used for qRT-PCR are listed in Table S2. Relative target gene expression was normalized to levels of the gapdh housekeeping gene, using the 2^−ΔΔCt^ methods.

### 2.3. Generation of Isogenic Gene Deletion Mutants and Complementation Strains

The construction of the transcription regulator gene knockout mutants and complementation strains was performed using a previously described procedure [23]. Briefly, DNA fragments corresponding to the upstream and downstream regions of the *SSUSC84_0170* gene were amplified using primer pairs 0170L1/0170L2 and 0170R1/0170R2. The two fragments were then merged by performing overlapping PCR [24]. The product was then cloned into the temperature-sensitive *S.suis*-*E.coli* shuttle vector pSET4s, yielding the knockout vector pSET4s:0170. The suspected mutant was verified by PCR using three pairs of primers: P1/P2 (to identify *gdh*), P3/P4 (to identify the spectinomycin resistance gene in the pSET4s vector), and 0170-1/0170-2 (to identify *SSUSC84_0170*). The other mutations were created in the same manner. The DNA fragment containing the *SSUSC84_0046* ORF and its promoter region fragment was amplified from the SC-19 genome. The fragment was cloned into pSET2 to form pSET2-*SSUSC84_0046*. The complementation strains (CΔ*0046*) were generated by electroporating the pSET2-*SSUSC84_0046* plasmid into Δ*0046*. The other complementation strains were created in the same manner. All primers are listed in Table S2.

### 2.4. Bacterial Growth Curves

Overnight cultures of the WT, mutation, and complementation strains were diluted 1:100 in fresh medium (tryptic soy broth with 10% newborn bovine serum) and incubated at 37 °C under appropriate conditions. Samples were taken from the cultures to measure the optical density at 600 nm (OD600) every hour.

### 2.5. Transmission Electron Microscopy

Transmission electron microscopy assays were performed in accordance with previously described methods with minor modifications [25]. WT, mutation, and complementation strains were harvested at an OD600 of 0.6 and fixed with 2.5% glutaraldehyde overnight. The samples were then treated with 2% osmium tetroxide for 2 h and dehydrated in a dilution series of ethanol. The dehydrated cells were embedded in epoxy resin and their morphological characteristics were observed using an H-7650 transmission electron microscope (Hitachi, Tokyo, Japan).

### 2.6. Experimental Infections of C57BL/6 Mice

For virulence studies, four-week-old female specific pathogen-free (SPF) C57BL/6 mice (10 mice per group) were challenged intraperitoneally with 200 μL of WT, mutant or complementation strains at approximately2.5 × 10^9^ CFU/mL in PBS or 3.5 × 10^9^ CFU/mL in PBS (5 × 10^8^ CFU for the Δ*0005*, Δ*0111* or Δ*0756* with the WT as a control and 7 × 10^8^ CFU for the remaining seven mutants with the WT and the complementation strains as a control). The infected mice were monitored for clinical signs and survival time for 7 days. The animals were euthanized at the end of the experimental period. All animal experiments were conducted in strict accordance with the recommendations of the China Regulations for the Administration of Affairs Concerning Experimental Animals (1988) and the Hubei Regulations for the Administration of Affairs Concerning Experimental Animals (2005). The protocol was approved by the Scientific Ethics Committee of Huazhong Agricultural University (approval number HZAUMO-2016-058).

### 2.7. In Vitro Bacterial Survival in the Presence of Mouse Whole Blood

Assays were performed in accordance with previously described methods with some modifications [26]. WT, mutants (Δ*comR*, Δ*sitR*, and Δ*sxvR*) and complementation strains (CΔ*comR*, CΔ*sitR*, and CΔ*sxvR*) were cultured to mid-log phase (OD600 of 0.6). The bacterial strains were then suspended in PBS. Subsequently, 900 μL of whole blood was mixed with 100 μL (10^4^ CFU) of SS2 cells and incubated for 2 h at 37 °C. The incubated mixtures were harvested at 0, 0.5, 1, and 2 h, serially diluted, vortexed, and plated on tryptic soy agar plates to determine bacterial survival. The assays were performed in triplicate and repeated three times.

### 2.8. Experimental Infections In Vivo

105 6-week-old female C57BL/6 mice were randomly assigned to seven groups with 15 mice/group and challenged intraperitoneally with a non-lethal dose (2 × 10^8^ CFU per mouse) of the WT, Δ*comR*, Δ*sitR*, Δ*sxvR*, CΔ*comR*, CΔ*sitR* and CΔ*sxvR* strains. At 3, 6, and 9 h post-infection, five mice per group were sacrificed to collect blood, which was used for bacteria counts and ELISAs to measure tumor necrosis factor-α (TNF-α), monocyte chemotactic protein-1 (MCP-1), and interleukin-1β (IL-1β) production.

### 2.9. RNA-Seq Analysis

To investigate gene expression profiles among Δ*comR*, Δ*sitR*, Δ*sxvR*, and WT strains, RNA-Seq was performed using Novogene (Tianjin, China). WT, Δ*comR*, Δ*sitR*, and Δ*sxvR* strains were cultured to mid-log phase (OD600 of 0.6). Six replicates were performed for each group, three of which were used for RNA-Seq and the remaining three were used to validate the results. The RNA extraction method was the same as previously described herein. Three biological replicates were mixed as one sample before RNA-Seq. RNA degradation and contamination were monitored on 1% agarose gels. RNA purity was checked using the NanoPhotometer^®^ spectrophotometer (IMPLEN, Westlake Village, CA, USA). RNA concentrations were measured using the Qubit^®^ RNA Assay Kit in Qubit^®^ 2.0 Fluorometer (Life Technologies, Carlsbad, CA, USA). The RNA integrity was assessed using the RNA Nano 6000 Assay Kit of the Bioanalyzer 2100 system (Agilent Technologies, Santa Clara, CA, USA). A library preparation for Transcriptome sequencing was assessed on the Agilent Bioanalyzer 2100 system after RNA quantification and qualification. The clustering of the index-coded samples was performed on a cBot Cluster Generation System using TruSeq PE Cluster Kit v3-cBot-HS (Illumina, San Diego, CA, United States) according to the manufacturer’s instructions. After cluster generation, the library preparations were sequenced on an Illumina Hiseq platform and 125-bp/150-bp paired-end reads were generated. The quality control of raw reads was performed and clean reads were mapped onto the complete reference *S.suis* SC84 genome. The FPKM (reads per kb per million reads) of each gene was calculated to consider the effect of sequencing depth and gene length on the read counts based on the length of the gene and read counts mapped to specific genes. Differential expression analysis was performed using the DESeq R package (1.18.0). The resulting *p*-values were adjusted using Benjamini and Hochberg’s approach for controlling the false discovery rate. Genes with an adjusted *p*-value < 0.05, as determined by DESeq, were assigned as differentially expressed. GO enrichment analysis of DEGs was implemented using the GOseq R package, in which gene length bias was corrected. GO terms with corrected *p*-values less than 0.05 were considered significantly enriched. We used KOBAS software to test the statistical enrichment of DEGs in KEGG pathways (http://www.genome.jp/kegg/).

### 2.10. Quantitative RT-PCR (qRT-PCR)

A subgroup of DEGs was selected to validate the RNA-Seq data based on SYBR green detection. Eight DEGs specific to Δ*comR* (*SSUSC84_0151*, *SSUSC84_0257*, *SSUSC84_0509*, *SSUSC84_0648*, *SSUSC84_1223*, *SSUSC84_1255*, *SSUSC84_1526*, and *SSUSC84_1726*) were chosen for qRT-PCR to confirm RNA-Seq data. For Δ*sitR*, *SSUSC84_0027*, *SSUSC84_0029*, *SSUSC84_0875*, *SSUSC84_0031*, *SSUSC84_1526*, *SSUSC84_1572*, *SSUSC84_1876*, and *SSUSC84_1927* and for Δ*sxvR*, *SSUSC84_0029*, *SSUSC84_0275*, *SSUSC84_0648*, *SSUSC84_0900*, *SSUSC84_1526*, *SSUSC84_1572*, *SSUSC84_ 1782* and *SSUSC84_1785* were selected. The primers (Table S2) were designed according to the genomic sequence of *S.suis* SC84. The RNA samples comprised the remaining three (of the six) replicates for each group prepared before RNA-Seq. qRT-PCR was performed using an ABI 7300HT Sequence Detection System using the ABI Power SYBR Green PCR Master Mix; gapdh served as an internal reference gene. The relative expression level was measured using the 2^−ΔΔCt^ methods. Data were reported as mean relative expression levels (± standard deviation) between WT and each mutant. Student’s t-tests were performed to verify the significance of real-time PCR quantifications.

### 2.11. Statistical Analysis

Statistics for the survival assay were done with the log-rank test (Mantel–Cox). Other statistical analyses were performed by one-way or two-way ANOVA, followed by a multiple comparison. All experiments were performed at least three times, and data are expressed as the mean ± SD. In the figures, * *p* < 0.05, ** *p* < 0.01, and *** *p* < 0.001. ns means no significance.

## 3. Results

### 3.1. Screening for Transcription Regulators by In vivo Induction

**Figure 1:**
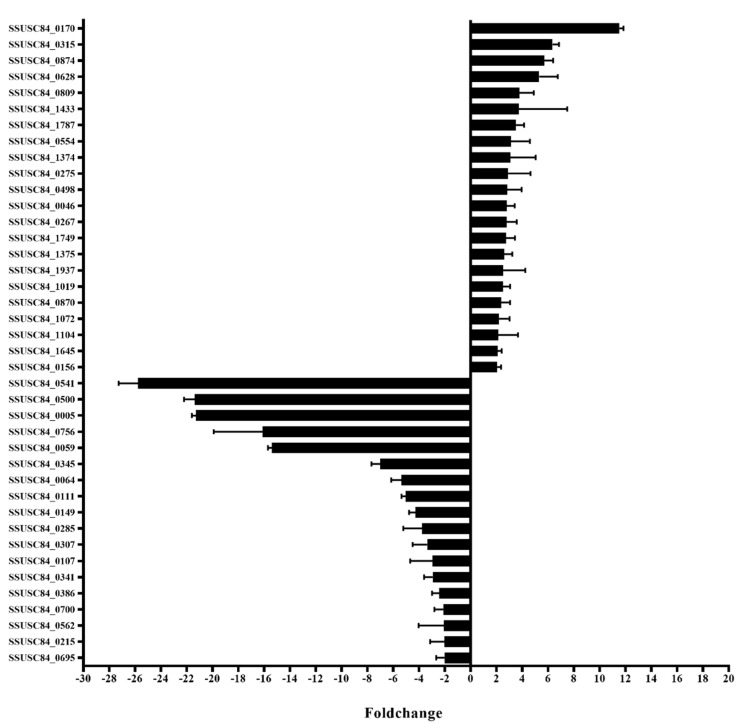
In vivo induction assay for the analysis of transcriptional regulators in the genome of *Streptococcus suis* SC84 that are differentially expressed (fold-change ≥ 2 or ≤ −2). Ten specific pathogen-free C57BL/6 mice were intraperitoneally infected with 5 × 10^8^ CFUs of the WT strain. Mice were sacrificed and dissected after 6 h. The spleens were harvested, and the bacteria were isolated. As a control, 5 mL of in vitro WT strains were collected when the OD was 0.6, representing mid-stationary phase. Furthermore, the bacterial RNA was extracted, and qRT-PCR was performed.

Transcription regulators were screened based on the annotation of protein domains in NCBI GenBank of SC84 (https://www.ncbi.nlm.nih.gov/nucleotide/251815212?report=genbank&log$=nuclalign&blast_rank=6&RID=U9GX5V1K014). With this, 89 hypothetical transcriptional regulators were predicted. Subsequently, qPCR was used to screen transcription regulators that were induced in vivo. The results indicated that 40 such factors were significantly differentially expressed between in vivo and in vitro conditions, of which 18 were down-regulated and 22 were up-regulated in vivo compared with in vitro (Figure 1).

### 3.2. Construction and Characterization of Mutants and Complementary Strains

**Figure 2:**
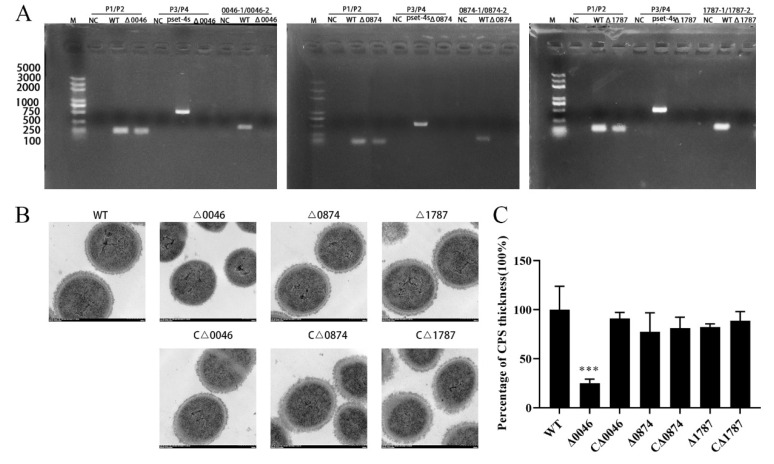
Construction of *Streptococcus suis* mutants and identification of phenotypes. (**A**) Confirmation of mutants by PCR using the primer pairs P1/P2 (to detect the *gdh* gene), P3/P4 (to detect the pSET4s vector), 0046-1/0046-2 (to detect the *SSUSC84_0046* gene), and 0874-1/0874-2 (to detect the *SSUSC84_0874* gene), 1787-1/1787-2 (to detect the *SSUSC84_1787* gene). (**B**) The capsules of WT, Δ*0046*, CΔ*0046*, Δ*0874*, CΔ*0874*, Δ*1787* and CΔ*1787* strains were detected by transmission electron microscopy (×20,000). (**C**) Image-pro Plus was used to measure and calculate the average capsular thickness. *** *p* < 0.001.

To investigate the roles of these transcriptional regulators in SS2 virulence, deletion mutants and their complementary strains were constructed. In total, 10 mutant strains (seven representing up-regulated and three representing down-regulated genes) were successfully obtained, and these were confirmed by PCR analysis. As a control, *gdh* could be detected in both WT and mutants. The spectinomycin resistance gene was detected in the pSET4s vector, but not in WT and mutant strains. At the same, every target gene was detected in the WT, but not in their respective deletion mutants. (Figure 2A and Figure S1A). In addition, growth curves indicated that growth in the mutants was not changed significantly compared to that in the WT and complementary strains [27].

Gram staining observations showed that the morphology and chain length of the mutants was also not significantly altered [27]. However, transmission electron microscopy results showed that the capsule of the Δ*0046* mutant was significantly thinner than that of the WT strain and CΔ*0046*. The measurement statistics showed that its thickness was about 25% of WT; however, there was no significant difference in capsular thickness between Δ*0874* or Δ*1787* and WT and their complementary strains (Figure 2B,C). Similarly, the capsules of other mutant strains did not differ significantly from WT (Figure S1B). This might indicate that regulator coding by *SSUSC84_0046* regulates the capsule of SS2.

### 3.3. ComR, sitR and sxvR Deletions Reduce S.suis Virulence in Mice

**Figure 3:**
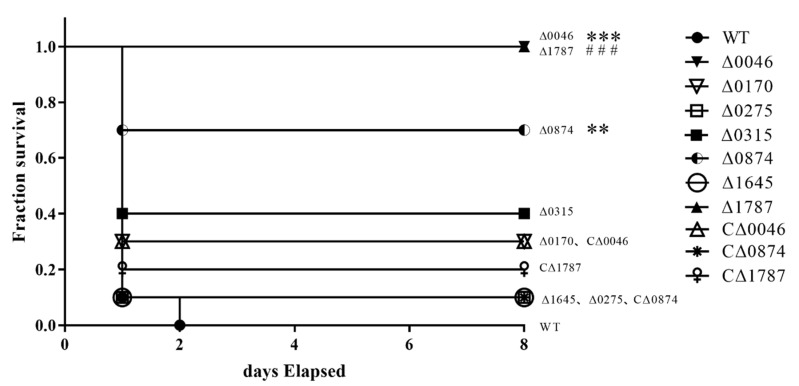
Survival curves of mice infected with SS2 strains. Female C57BL/6 mice in different groups were challenged intraperitoneally with 7 × 10^8^ colony-forming units of WT, Δ*0046*, Δ*0170*, Δ*0275*, *0315*, Δ*0874*, Δ*1645*, Δ*1787*, CΔ*0046*, CΔ*0874* or CΔ*1787* strains cultured on tryptic soy agar. The mortality of mice was recorded for 7 days. The results shown are representative of three independent experiments. ** *p* < 0.01 for Δ*0874* versus WT; *** *p* < 0.001 for Δ*0046* versus WT; ### *p* < 0.001 for Δ*1787* versus WT.

To evaluate the individual effects of deleting 10 transcriptional regulators on *S.suis* virulence, mouse infection experiments were performed. Since *SSUSC84_0005*, *SSUSC84_0111*, and *SSUSC84_0756* were down-regulated in vivo, these mutants may lead to increased virulence, so we performed mouse experiments with a dose of half the lethal dose (5 × 10^8^ CFU). However, the results showed there was no significant change in virulence between the mutants and WT (Figure S2). The results showed that mice infected with the WT strain died within 2 days. However, the group infected with Δ*0046* or Δ*1787* strains all survived after 7 days without obvious symptoms and seven mice survived after 1 week upon infection with the Δ*0874* strain. Although some disease signs appeared shortly after infection, animals quickly recovered. The mortality of mice infected with complementary strains CΔ*0046*, CΔ*0874* or CΔ*1787* was 70, 90 and 80%, respectively, which showed no significant difference with the mortality of WT (100%). Furthermore, mice infected with the other seven mutant strains all died within 2 days. Based on statistical analysis, we initially concluded that the virulence of Δ*0046*, Δ*1787*, and Δ*0874* mutants was significantly reduced (Figure 3, ** *p* < 0.01 for Δ*0874* versus WT, *** *p* < 0.001 for Δ*0046* versus WT, ### *p* < 0.001 for Δ*1787* versus WT). According to the relevant homologous genes, we named these three transcriptional regulators *comR* (*SSUSC84_0046*), *sitR* (*SSUSC84_0874*), and *sxvR* (*SSUSC84_1787*), respectively, for further study.

### 3.4. The Domains of comR, sitR, and sxvR Indicate That These Three Proteins Are Transcription Regulators

The *comR* gene contained 900 nucleotides. Genomic BLAST comparison showed that it was widespread in *S. suis* and was highly conserved in 58 strains of *S. suis* that had been completely sequenced. The conserved domain of the protein was analyzed by BLASTp program. The result showed that there was a helix-turn-helix DNA-binding domain at the N-terminus, which was a common feature among members of the xenobiotic response element family [28] (Figure S3A). The *sitR* gene contained 591 nucleotides. Genomic BLAST comparison showed that this gene was widespread in the virulent strains of *S. suis*, and was highly conservative in other *Streptococcus* species, such as *S. thermophilus*, *S. anlactococcus*, *S. Anginosus*, etc. The protein of *sitR* contained two domains. The 1–97 bp at the N-terminus was the COG5340 domain, which was the domain of the transcription factor in the process of bacterial resistance to viruses and 5–51bp at the N-terminus contained the AbiEi_4 domain, which was related to the transcriptional regulator of the cognate antitoxin of the type IV toxin-antitoxin “innate immunity” bacterial abortive infection (Abi) system (Figure S3B). *SxvR* contained 1038 nucleotides. Similar to *comR*, *sxvR* was also widely found in *S. suis*, and was highly conserved in the 58 completely sequenced genomes of *S. suis*. The protein of *sxvR* contained a PRK08099 domain (1–341) which was related to bifunctional DNA-binding transcriptional repressor/NMN adenylyltransferase (Figure S3C). The results of gene alignment and protein domain prediction indicate that these proteins are transcriptional regulators and are very conserved in *S. suis.*

### 3.5. Survival Ability of Mutants in Whole Blood is Diminished

**Figure 4:**
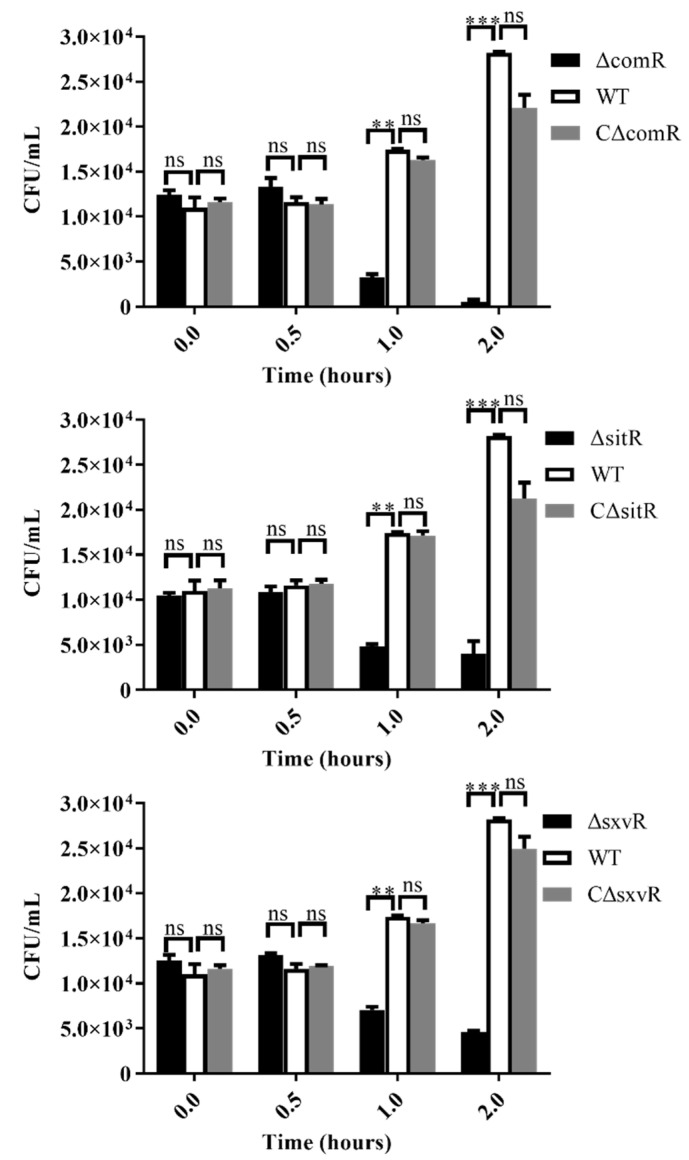
In vitro bacterial survival in the presence of mouse whole blood. SS2 WT, Δ*comR*, CΔ*comR*, Δ*sitR*, CΔ*sitR*, Δ*sxvR*, and CΔ*sxvR* stains were cultured to mid-log phase (OD600 of 0.6). Bacteria were then suspended in PBS, and subsequently, 900 μL of whole blood was mixed with 100 μL of SS2 cells and incubated for 2 h at 37 °C. The incubated mixtures were harvested at 0, 0.5, 1 and 2 h, serially diluted, vortexed, and plated on tryptic soy agar plates to determine the number of bacteria. The assays were performed in triplicate and repeated three times. ** *p* < 0.01, *** *p* < 0.001, ns means no significance.

To model the ability of each mutant to escape the immune system, we tested the growth of Δ*comR*, Δ*sitR*, and Δ*sxvR* mutants in whole blood collected from C57BL/6 mice and used the WT and complementary strains as controls. The initial bacterial amount at 0 h was 10^4^ CFU. Results showed no significant differences between mutants and WT strains at 0.5 h (mean amount for Δ*comR*, Δ*sitR*, Δ*sxvR*, and WT strains were 1.33 × 10^4^,1.09 × 10^4^,1.32 × 10^4^ and 1.17 × 10^4^ CFU respectively). However, after 1 and 2 h, the growth rate of the mutant was significantly slower than that of the WT (mean amount for Δ*comR*, Δ*sitR*, Δ*sxvR*, and WT strains were 3.25 × 10^3^, 4.8 × 10^3^, 7.05 × 10^3^ and 1.74 × 10^4^ CFU, respectively, at 1 h and 5.0 × 10^2^,4.0 × 10^3^,4.6 × 10^3^, and 2.82 × 10^4^, respectively, at 2 h). The mean amounts of CΔ*comR*, CΔ*sitR* and CΔ*sxvR* were similar to WT at the three time points (Figure 4).

### 3.6. ΔcomR, ΔsitR, and ΔsxvR Mutants Exhibit Decreased Pro-Inflammatory Ability and Reduced Bacterial Loads in Mice

**Figure 5:**
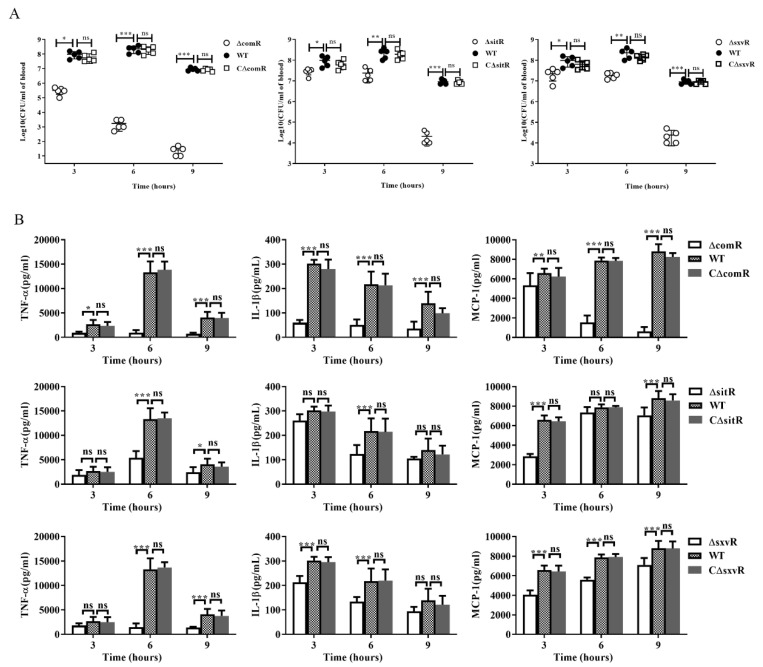
Bacterial loads and cytokine concentrations in the blood of mice after SS2 infection. Female C57BL/6 mice were challenged with 2 × 10^8^ CFU of WT, Δ*comR*, CΔ*comR*, Δ*sitR*, CΔ*sitR*, Δ*sxvR* or CΔ*sxvR* strains cultured on tryptic soy agar. After infection for 3, 6, or 9 h, an equal number of mice in each group were sacrificed to collect blood. (**A**) Bacteria loads in the blood were examined by determining CFU counts. The results shown are representative of three independent experiments. (**B**) Blood cytokine concentrations were determined by enzyme-linked immunosorbent assay. * *p* < 0.05, ** *p* < 0.01, *** *p* < 0.001, ns means no significance.

To further analyze the mechanism through which deletion of *comR*, *sitR* or *sxvR* results in attenuated virulence, C57BL/6 mice were challenged intraperitoneally with a non-lethal dose of these strains, their complementary strains or the WT, and blood was collected to determine bacterial loads and cytokine concentrations. The results showed that bacterial loads of Δ*comR*, Δ*sitR*, and Δ*sxvR* mutants were all significantly lower than that of the WT at 3, 6, and 9 h, which was especially true for Δ*comR*, in which only few bacteria (4% of the initial bacterial volume) were detected after 9 h (Figure 5A). At the same time, cytokine levels in the blood of animals infected with these three mutants also decreased to different degrees. Specifically, attenuation in the Δ*comR* group was the most obvious as the concentrations of TNF-a, IL-1β, and MCP-1 were all significantly reduced at 3, 6, and 9 h post-infection (Figure 5B). Overall, our data suggested that these three transcriptional regulators might contribute to SS2 virulence by inducing heightened host pro-inflammatory responses and affecting in vivo bacterial loads.

### 3.7. Differentially Expressed Genes (DEGs) in Mutant Strains

To understand the transcriptomic dysregulation that occurs in the three mutants, gene expression profiles were determined by RNA-Seq, compared to those of the WT control. Expression levels of 753 genes were altered in the Δ*comR* mutant. Among them, 330 were up-regulated and 423 were down-regulated (*p* < 0.05, fold change > 1.5). These genes were found to be involved in diverse biological processes including ABC transporters and metabolism (Table S3). Furthermore, based on the same standards, for Δ*sitR* and Δ*sxvR* mutants, there were 23 (22 down-regulated and 1 up-regulated) and 56 (35 down-regulated and 21 up-regulated) DEGs. Among these were genes involved in nucleotide metabolism, biosynthesis of secondary metabolites, and amino acid metabolism (Table S3). The original data of RNA-Seq has been uploaded to SRA database of NCBI with ID SUB6386663.The results of RNA-Seq were verified by qRT-PCR. Eight genes with various expression levels in each mutant were selected for qRT-PCR analysis and the correlations between the two methods were all very high (R^2^ for Δ*comR*, Δ*sitR*, and Δ*sxvR* were 0.9150, 0.9304, and 0.9533, respectively; Figure S4).

### 3.8. Functional Analysis of Virulence-Associated Genes (VAGs) and DEGs

RNA-Seq results demonstrated a change in the expression of many genes involved in virulence or those correlated with virulence. We compared DEGs using the virulence factor database (http://www.mgc.ac.cn/VFs/search_VFs.htm) and known VAGs of *S.suis* [6,29]. The expression levels of 184 virulence factors or virulence-related factors (60 up-regulated and 124 down-regulated) in the *comR* deletion mutant were found to be altered (Table S4; fold-change > 1.5, *p* < 0.05). To further analyze the roles of these DEGs, KEGG and Gene Ontology (GO)-term analysis was performed. Four pathways were repressed with the *comR*-deletion including ABC transporters (sss02010), amino acid metabolism (sss00350 and sss00330), biotin metabolism (sss00780), and fatty acid metabolism (sss01212 and sss00061) (Table 1). GO-term results showed that these genes were assigned to various classes including cellular component, membrane proteins, transport and so on (Figure S5A). Capsular polysaccharide assembly related genes including the capsule synthesis genes *cps2E* (0.60-fold), *cps2F* (0.59-fold), *cps2G* (0.53-fold), *cps2H* (0.51-fold), *cps2J* (0.43-fold), and *cps2K* (0.47-fold) and sialic acid synthesis genes including *neuA* (0.2-fold), *neuB* (0.3-fold), and *neuC* (0.3-fold) were significantly down-regulated in the Δ*comR* mutant compared to expression in the WT. Since the capsule is an important virulence factor for *S.suis*, *comR* might regulate virulence mainly by regulating the capsule. Furthermore, the expression of other genes that have been reported to be virulence factors in SS2 was also reduced. These include surface-anchored DNA nuclease (*ssnA*, 0.37-fold), putative surface-anchored protein (*sao*, 0.34-fold), response regulator protein of the SalRK TCS (*SalR*, 0.66-fold), and putative pilus subunit protein (*SBP2*, 0.25-fold; Table 2).

Furthermore, there were nine virulence factors or virulence-related factors in the virulence factor database that were down-regulated after deletion of the *sitR* gene (Table S4; fold-change > 1.5, *p* < 0.05). Three pathways were repressed with the *sitR*-deletion including purine metabolism (sss00230), biosynthesis of secondary metabolites (sss01110) and one carbon pool by folate (sss00670) (Table 1). GO-term results showed that these genes were mainly involved in small molecule metabolic (Figure S5B). There were three genes that had been reported in *S.suis*, including *argR* (0.65-fold), *dpr* (0.60-fold), and *purD*, (0.62-fold) (Table 2). No genes associated with virulence up-regulation were found. This might suggest that *sitR* influences virulence in vivo through the positive regulation of virulence factors. Similarly, the absence of *sxvR* resulted in changes in the expression of 11 virulence factors (Table S4; fold-change > 1.5, *p* < 0.05), of which five were up-regulated and six were down-regulated. Four pathways were repressed with the *sxvR*-deletion including purine metabolism (sss00230), biosynthesis of secondary metabolites (sss01110), one carbon pool by folate (sss00670) and Alanine, aspartate and glutamate metabolism (Table 1). GO-term results showed that these virulence factors genes mainly involve into nucleobase-containing small molecule metabolic process, organonitrogen compound metabolic process and nucleobase-containing small molecule metabolic process (Figure S5C). There were two genes that have been reported in *S.suis*, including *ssnA* (0.61-fold) and NADH oxidase (*nox*, 0.53-fold), which were previously reported as virulence-related factors (Table 2).

## 4. Discussion

To date, *S.suis* remains a huge threat to the swine industry and human health [12]. Although some virulence-related factors have been reported in recent years [6], research on virulence-related transcriptional regulators is poor. Furthermore, there is almost no research on the screening of virulence-related transcription regulators at the entire genome level. In this study, we started from the entire genome of *S.suis* and screened all transcriptional regulatory genes. The screening method of transcription regulator was based on the gene annotations in NCBI, which were obtained through the special domains of transcription regulator, such as helix-turn-helix (HTH), leucine zipper, zinc finger structure, etc. However, there were many hypothesized proteins on the genome that had not been annotated, and these proteins may also contain transcription regulators. In addition, the mutants of some selected up-regulated transcription regulators in vivo were not successfully constructed. Therefore, further research is needed in the future. By performing large-scale screening for changes in vivo expression, many factors that might be associated with virulence were identified. This method was previously used to screen for novel virulence factors in Listeria monocytogenes [20] and was first used here to identify virulence-related transcriptional regulators in *S.suis*. Through in vivo-induced, mutant construction, and animal experiments, *SSUSC84_0046*, *SSUSC84_0874*, and *SSUSC84_1787* were found to be involved in the regulation of virulence. *SSUSC84_0046* was previously reported in *S.suis* as *comR*, which is involved in the regulation of competence for DNA transformation [30]. However, there were no previous reports of *SSUSC84_0874* and *SSUSC84_1787* homologs, and thus we named them *sitR* and *sxvR*.

Proper levels of cytokines during the inflammatory response have an important effect on the host defense against pathogens and the clearance of bacterial infections. However, overexpression of cytokines can lead to organ damage and exacerbate disease progression [12,31]. Furthermore, streptococcal toxic shock-like syndrome, characterized by high bacterial loads and enhanced inflammation, is an important feature of *S.suis* infection, and sometimes leads to the rapid death of infected animals [32]. To further study the effect of three transcriptional regulators on virulence, we measured in vitro growth in whole-blood samples, in vivo bacterial loads, and cytokine expression measurements. As expected, all three mutants had significantly reduced viability in whole blood in vitro and in vivo and suppressed cytokine production to varying degrees. Among these mutants, the phenotype of Δ*comR* was particularly obvious (Figure 4 and Figure 5). These results showed that the decrease in virulence upon deletion of these three regulators might be attributed to not only a decline in bacterial numbers but also to inhibition of the inflammatory effect after infection. In this study, we used mice as an infection model. Although the natural host of *S.suis* is pig, the mouse model of *S.suis* infection has been widely used, which reproduce similar clinical signs to those observed during the systemic (septic shock) and CNS (meningitis) infections in the natural host [33].

*ComR* was previously reported to be involved in natural competence for the uptake of extracellular DNA and genetic transformation in streptococci [34]. It was first identified in *S.suis* by Zaccaria et al. [30], and later these authors studied the partial mechanism of activation. Simply, extracellular sigX-inducing-peptide (XIP) enters the bacteria via the opp transporter system. After that, *comR* binds to XIP, and the *comR*–XIP complex promotes the expression of *comX*, which activates the expression of the late-competence genes involved in the transformasome [35]. Furthermore, Sigma X enhances the expression of *comR* resulting in a positive feedback loop [36].

In our study, we found that *comR* was also involved in the regulation of virulence-related genes. Deletion of *comR* resulted in the altered expression of different virulence-related genes including those involved in metabolism and ABC transporters. Among these, capsular polysaccharide assembly related gene clusters including capsule synthesis genes (*cps2E-2K*) and sialic acid synthesis genes (*neuA/B/C*) were significantly down-regulated and this result was consistent with that of transmission electron microscopy (Figure 2B,C). CPS has been proven to be a critical virulence factor in SS2. It can help the pathogen resist neutrophil-and monocyte/macrophage-mediated phagocytosis and killing, which ensures survival in the blood [6]. This result was consistent with the experiment of survival in the blood in vitro and vivo (Figure 4 and Figure 5A). The N-acetylneuraminic acid (sialic acid) linked to the end of the CPS chain also plays a role in SS2 virulence [37,38]. These results thus indicate that *comR* might affect virulence by regulating capsule synthesis. In addition, the expression of other reported virulence factors was also reduced. These include genes of the surface-anchored DNA nuclease (*ssnA*) [11,39] and response regulator protein of the SalR/K TCS (*SalR*) [40] (Table 2). Moreover, ABC transporters play various roles in *S.suis*, many of which are involved in virulence, such as host colonization and biofilm formation [41,42]. In addition, previous reports have shown that *comR* up-regulation leads to elevated expression of the pilus, which allows exogenous DNA to be internalized into the cytoplasm in other *Streptococcus* groups [35,43]. In the present study, pilus-associated genes were down-regulated after *comR* was deleted. This was consistent with previous research results. Furthermore, pilus genes are also considered to be involved in virulence [44]. These facts indicate that *comR* may be a global regulatory factor which regulates virulence through complicated mechanisms, in addition to regulating the capsule.

The genes that were altered after *sitR* deletion were almost all down-regulated, and we identified some potential virulence and regulatory factors. *argR* can catalyze the conversion of arginine to ornithine, ammonia and carbon dioxide to increase the pH in the acidic environment [4,45]. Moreover, *dpr* knockout strains are highly sensitive to H_2_O_2_ [46]. As acidic and oxidative environments are important ways for the host immune system to clear bacteria [6], these suggest that the ability of *S.suis* to resist the harsh environment of its host decreased after the loss of *sitR*. In addition, a Δ*PurM* mutant was unable to replicate in human cells and was completely avirulent in C57BL/6 mice following high-dose intranasal inoculation [47]. This prompts us to suggest that *sitR* might affect virulence by regulating these virulence factors, but whether the regulation of these virulence factors is direct or indirect requires further verification. The expression of *ssnA* and *nox* was also significantly reduced after deletion of *sxvR*. *SsnA* is a previously discovered cell wall-anchored extracellular DNase, which is important for neutrophil extracellular trap (NET) degradation; deletion of *ssnA* resulted in a significant attenuation of virulence when evaluated in CD1 mice [39,48]. Furthermore, the Δ*nox* strain exhibited reduced tolerance to oxidative stress and *nox* was found to contribute to the virulence of *S.suis* in a murine infection model [49]. These results suggest that the decreased virulence with *svxR* deletion is caused by a decline in the ability to evade the host immune system.

In conclusion, we screened three transcriptional regulators that affect virulence through in vivo induction, mutant construction, and animal experiments. The regulatory mechanism of these transcriptional regulators and the initial mechanism of their regulation during virulence were analyzed by RNA-Seq. Based on the results, it appears that the results of this study present three novel virulence-related transcriptional regulators for *S.suis*. The information could be used to further define regulatory networks associated with the pathogenesis of this organism. Furthermore, these factors could be targeted to control infection associated with *S.suis*.

## Figures and Tables

**Table 1:** Pathways repressed in the Δ*comR*.

Pathway	ID	Input Number	Background Number	*p*-Value
Δ*comR* vs. WT				
ABC transporters	sss02010	28	75	5.76 × 10^−13^
Tyrosine metabolism	sss00350	3	5	0.009213
Biotin metabolism	sss00780	3	5	0.009213
Fatty acid metabolism	sss01212	4	12	0.012373
Fatty acid biosynthesis	sss00061	4	13	0.015451
Arginine and proline metabolism	sss00330	3	12	0.055231
Δ*sitR* vs. WT				
Purine metabolism	sss00230	8	51	1.487 × 10^−6^
Biosynthesis of secondary metabolites	sss01110	7	152	0.0087595
One carbon pool by folate	sss00670	2	10	0.014002
Δ*sxvR* vs. WT				
Purine metabolism	sss00230	6	51	0.0006233
One carbon pool by folate	sss00670	2	10	0.0216202
Biosynthesis of secondary metabolites	sss01110	7	152	0.030136
Alanine, aspartate and glutamate metabolism	sss00250	2	15	0.0419204

**Table 2:** Virulence-associated factor (VFG) genes in *S. suis* Δ*comR*, Δ*sitR*, and Δ*sxvR* mutants, as identified by RNA-Seq.

Gene_id	Genename	Foldchange (log2)	Padj	Description
Δ*comR* vs. WT				
*SSUSC84_0504*	*cps2E*	−0.7278	6.7712 × 10^−47^	putative galactosyl transferase
*SSUSC84_0505*	*cps2F*	−0.75585	4.5179 × 10^−50^	putative rhamnosyl transferase
*SSUSC84_0506*	*cps2G*	−0.9129	7.2375 × 10^−72^	putative glycosyl transferase
*SSUSC84_0507*	*cps2H*	−0.96001	1.2376 × 10^−79^	putative membrane protein
*SSUSC84_0509*	*cps2J*	−1.1937	4.035 × 10^−115^	putative glycosyltransferase
*SSUSC84_0510*	*cps2K*	−1.0932	1.3498 × 10^−96^	putative glycosyl transferase
*SSUSC84_1270*	*ftsX*	−0.8355	1.9701 × 10^−38^	putative cell division protein
*SSUSC84_0648*	*nox*	−0.99753	1.7347 × 10^−09^	NADH oxidase
*SSUSC84_1782*	*ssnA*	−1.4199	1.0774 × 10^−85^	surface-anchored DNA nuclease
*SSUSC84_1234*	*sao*	−1.5613	1.3274 × 10^−210^	putative surface-anchored protein
*SSUSC84_0520*	*neuA*	−1.7595	1.6735 × 10^−254^	N-acylneuraminate cytidylyltransferase
*SSUSC84_0517*	*neuB*	−1.5933	3.3455 × 10^−195^	putative N-acetylneuraminic acid synthase
*SSUSC84_0518*	*neuC*	−1.6449	2.7075 × 10^−214^	putative UDP-N-acetylglucosamine 2-epimerase
*SSUSC84_1502*	*ofs*	−2.3267	0	serum opacity factor
*SSUSC84_1907*	*SBP2*	−2.0237	3.44 × 10^−236^	putative accessory pilus subunit
*SSUSC84_0849*	*SalR*	−0.59813	0.00049797	response regulator protein
*SSUSC84_1566*	*csrR*	−0.59471	1.5809 × 10^−17^	response regulator protein
Δ*sitR* vs. WT				
*SSUSC84_1927*	*argR*	−0.61878	0.00013602	putative arginine repressor
*SSUSC84_1526*	*dpr*	−0.74885	0.020193	Dps-like peroxide resistance protein Dpr
*SSUSC84_0031*	*purD*	−0.68965	0.00043377	phosphoribosylamine-glycine ligase
Δ*sxvR* vs. WT				
*SSUSC84_0648*	*nox*	−0.91682	2.2094 × 10^−7^	NADH oxidase
*SSUSC84_1782*	*ssnA*	−0.71857	3.5286 × 10^−25^	surface-anchored DNA nuclease

## Supplementary Materials

The following are available online at https://www.mdpi.com/2073-4425/11/9/972/s1, Figure S1: Construction of other SS2 mutants and identification of phenotypes. (A) Confirmation of mutants by PCR using the primer pairs P1/P2 (to detect the *gdh* gene), P3/P4 (to detect the pSET4s vector), 0005-1/0005-2 (to detect the *SSUSC84_0005* gene), and 0111-1/0111-2 (to detect the *SSUSC84_0111* gene), 0170-1/0170-2 (to detect the *SSUSC84_0170* gene), 0275-1/0275-2 (to detect the *SSUSC84_0275* gene), 0315-1/0315-2 (to detect the *SSUSC84_0315* gene), 0756-1/0756-2 (to detect the *SSUSC84_0756* gene), 1645-1/1645-2 (to detect the *SSUSC84_1645* gene). (B) The capsules of WT, Δ*0005*, Δ*0111*, Δ*0170*, Δ*0275*, Δ*0315*, Δ*0756*, and Δ*1645* strains were detected by transmission electron microscopy (×20,000). Figure S2: Survival curves of mice infected with SS2 strains. Female C57BL/6 mice in different groups were challenged intraperitoneally with 5 × 10^8^ colony-forming units of WT, Δ*0005*, Δ*0111*, or Δ*0756* strains cultured on tryptic soy agar. The mortality of mice was recorded for 7 days. The results shown are representative of three independent experiments. Figure S3: Domains of the three proteins (A) *comR* (B) *sitR* and (C) *sxvR*. Figure S4: Verification of RNA sequencing results. Figure S5: The most enriched GO terms based on analyses of VFG genes. The down-regulated DEGs of the mutant strains were compared to the virulence factor database and known VAGs of *S.suis.* Then the selected genes were analyzed by GO terms. (A) Δ*comR* (B) Δ*sitR* (C) Δ*sxvR*. Table S1: Summary of bacterial strains and plasmid used in this study. Table S2: Oligonucleotide primers used in this study. Table S3: KEGG analyses of the pathways involved in the DEGs of Δ*comR*, Δ*sitR* and Δ*sxvR*. Table S4: VFG genes in △*comR*, △*sitR* and △*sxvR* identified by RNA-Seq.

## References

[1] Zheng F., Ji H., Cao M., Wang C., Feng Y., Li M., Pan X., Wang J., Qin Y., Hu F. (2011). Contribution of the Rgg transcription regulator to metabolism and virulence of *Streptococcus suis* serotype 2. Infect. Immun..

[2] Zhao J., Lin L., Fu L., Han L., Zhang A. (2016). Neutrophil extracellular Traps play an important role in clearance of *Streptococcus suis* in vivo. Microbiol. Immunol..

[3] Filippitzi M.E., Goumperis T., Robinson T., Saegerman C. (2017). Microbiological zoonotic emerging risks, transmitted between livestock animals and humans (2007–2015). Transbound. Emerg. Dis..

[4] Dutkiewicz J., Zajac V., Sroka J., Wasinski B., Cisak E., Sawczyn A., Kloc A., Wojcik-Fatla A. (2018). *Streptococcus suis*: A re-emerging pathogen associated with occupational exposure to pigs or pork products. Part II-Pathogenesis. Ann. Agric. Environ. Med..

[5] Tien le H.T., Nishibori T., Nishitani Y., Nomoto R., Osawa R. (2013). Reappraisal of the taxonomy of *Streptococcus suis* serotypes 20, 22, 26, and 33 based on DNA-DNA homology and sodA and recN phylogenies. Vet. Microbiol..

[6] Fittipaldi N., Segura M., Grenier D., Gottschalk M. (2012). Virulence factors involved in the pathogenesis of the infection caused by the swine pathogen and zoonotic agent *Streptococcus suis*. Future Microbiol..

[7] Zhao J., Pan S., Lin L., Fu L., Yang C., Xu Z., Wei Y., Jin M., Zhang A. (2015). *Streptococcus suis* serotype 2 strains can induce the formation of neutrophil extracellular traps and evade trapping. FEMS Microbiol. Lett..

[8] Segura M., Fittipaldi N., Calzas C., Gottschalk M. (2017). Critical *Streptococcus suis* virulence factors: Are they all really critical?. Trends Microbiol..

[9] Zhang Y., Zong B., Wang X., Zhu Y., Hu L., Li P., Zhang A., Chen H., Liu M., Tan C. (2018). Fisetin Lowers *Streptococcus suis* serotype 2 pathogenicity in mice by inhibiting the hemolytic activity of suilysin. Front. Microbiol..

[10] Gottschalk M.G., Lacouture S., Dubreuil J.D. (1995). Characterization of *Streptococcus suis* capsular type 2 haemolysin. Microbiology.

[11] de Buhr N., Neumann A., Jerjomiceva N., von Kockritz-Blickwede M., Baums C.G. (2014). *Streptococcus suis* DNase SsnA contributes to degradation of neutrophil extracellular traps (NETs) and evasion of NET-mediated antimicrobial activity. Microbiology.

[12] Zhang Q., Huang J., Yu J., Xu Z., Liu L., Song Y., Sun X., Zhang A., Jin M. (2017). HP1330 contributes to *Streptococcus suis* virulence by inducing toll-like receptor 2- and ERK1/2-dependent pro-inflammatory responses and influencing in vivo *S. suis* loads. Front. Immunol..

[13] Goyette-Desjardins G., Auger J.P., Xu J., Segura M., Gottschalk M. (2014). *Streptococcus suis*, an important pig pathogen and emerging zoonotic agent-an update on the worldwide distribution based on serotyping and sequence typing. Emerg. Microbes Infect..

[14] Houde M., Gottschalk M., Gagnon F., Van Calsteren M.R., Segura M. (2012). *Streptococcus suis* capsular polysaccharide inhibits phagocytosis through destabilization of lipid microdomains and prevents lactosylceramide-dependent recognition. Infect. Immun..

[15] Benga L., Goethe R., Rohde M., Valentin-Weigand P. (2004). Non-encapsulated strains reveal novel insights in invasion and survival of *Streptococcus suis* in epithelial cells. Cell. Microbiol..

[16] Tan C., Liu M., Jin M., Liu J., Chen Y., Wu T., Fu T., Bei W., Chen H. (2008). The key virulence-associated genes of *Streptococcus suis* type 2 are upregulated and differentially expressed in vivo. FEMS Microbiol. Lett..

[17] Beier D., Gross R. (2006). Regulation of bacterial virulence by two-component systems. Curr. Opin. Microbiol..

[18] Han H., Liu C., Wang Q., Xuan C., Zheng B., Tang J., Yan J., Zhang J., Li M., Cheng H. (2012). The two-component system Ihk/Irr contributes to the virulence of *Streptococcus suis* serotype 2 strain 05ZYH33 through alteration of the bacterial cell metabolism. Microbiology.

[19] Chen W.J., Zhu T. (2004). Networks of transcription factors with roles in environmental stress response. Trends Plant Sci..

[20] Camejo A., Buchrieser C., Couve E., Carvalho F., Reis O., Ferreira P., Sousa S., Cossart P., Cabanes D. (2009). In vivo transcriptional profiling of *Listeria monocytogenes* and mutagenesis identify new virulence factors involved in infection. PLoS Pathog..

[21] Milohanic E., Glaser P., Coppee J.Y., Frangeul L., Vega Y., Vazquez-Boland J.A., Kunst F., Cossart P., Buchrieser C. (2003). Transcriptome analysis of *Listeria monocytogenes* identifies three groups of genes differently regulated by PrfA. Mol. Microbiol..

[22] Moore L.J., Pridmore A.C., Lee M.E., Read R.C. (2005). Induction of pro-inflammatory cytokine release by human macrophages during exposure of *Streptococcus pneumoniae* to penicillin is influenced by minimum inhibitory concentration ratio. Int. J. Antimicrob. Agents.

[23] Takamatsu D., Osaki M., Sekizaki T. (2001). Thermosensitive suicide vectors for gene replacement in *Streptococcus suis*. Plasmid.

[24] Warrens A.N., Jones M.D., Lechler R.I. (1997). Splicing by overlap extension by PCR using asymmetric amplification: An improved technique for the generation of hybrid proteins of immunological interest. Gene.

[25] Gao T., Tan M., Liu W., Zhang C., Zhang T., Zheng L., Zhu J., Li L., Zhou R. (2016). GidA, a tRNA modification enzyme, contributes to the growth, and virulence of *Streptococcus suis* serotype 2. Front. Cell. Infect. Microbiol..

[26] Pian Y., Gan S., Wang S., Guo J., Wang P., Zheng Y., Cai X., Jiang Y., Yuan Y. (2012). Fhb, a novel factor H-binding surface protein, contributes to the antiphagocytic ability and virulence of *Streptococcus suis*. Infect. Immun..

[27] Liu L. (2020).

[28] Barragan M.J., Blazquez B., Zamarro M.T., Mancheno J.M., Garcia J.L., Diaz E., Carmona M. (2005). BzdR, a repressor that controls the anaerobic catabolism of benzoate in Azoarcus sp. CIB, is the first member of a new subfamily of transcriptional regulators. J. Biol. Chem..

[29] Zhang C., Sun W., Tan M., Dong M., Liu W., Gao T., Li L., Xu Z., Zhou R. (2017). The eukaryote-like serine/threonine kinase STK regulates the growth and metabolism of zoonotic *Streptococcus suis*. Front. Cell. Infect. Microbiol..

[30] Zaccaria E., van Baarlen P., de Greeff A., Morrison D.A., Smith H., Wells J.M. (2014). Control of competence for DNA transformation in *Streptococcus suis* by genetically transferable pherotypes. PLoS ONE.

[31] Tisoncik J.R., Korth M.J., Simmons C.P., Farrar J., Martin T.R., Katze M.G. (2012). Into the eye of the cytokine storm. Microbiol. Mol. Biol. Rev..

[32] Dominguez-Punaro M.C., Segura M., Plante M.M., Lacouture S., Rivest S., Gottschalk M. (2007). *Streptococcus suis* serotype 2, an important swine and human pathogen, induces strong systemic and cerebral inflammatory responses in a mouse model of infection. J. Immunol..

[33] Auger J.P., Fittipaldi N., Benoit-Biancamano M.O., Segura M., Gottschalk M. (2016). virulence studies of different sequence types and geographical origins of *Streptococcus suis* serotype 2 in a mouse model of infection. Pathogens.

[34] Mashburn-Warren L., Morrison D.A., Federle M.J. (2010). A novel double-tryptophan peptide pheromone controls competence in Streptococcus spp. via an Rgg regulator. Mol. Microbiol..

[35] Zaccaria E., Wels M., van Baarlen P., Wells J.M. (2016). Temporal regulation of the transformasome and competence development in *Streptococcus suis*. Front. Microbiol..

[36] Khan R., Junges R., Amdal H.A., Chen T., Morrison D.A., Petersen F.C. (2017). A positive feedback loop mediated by Sigma X enhances expression of the streptococcal regulator ComR. Sci. Rep..

[37] Zhong X., Zhang Y., Zhu Y., Dong W., Ma J., Pan Z., Roy S., Lu C., Yao H. (2018). The two-component signaling system VraSRSS is critical for multidrug resistance and full virulence in *Streptococcus suis* serotype 2. Infect. Immun..

[38] Smith H.E., Damman M., van der Velde J., Wagenaar F., Wisselink H.J., Stockhofe-Zurwieden N., Smits M.A. (1999). Identification and characterization of the cps locus of *Streptococcus suis* serotype 2: The capsule protects against phagocytosis and is an important virulence factor. Infect. Immun..

[39] Li M., Cai R.J., Li C.L., Song S., Li Y., Jiang Z.Y., Yang D.X. (2017). Deletion of ssnA attenuates the pathogenicity of *Streptococcus suis* and confers protection against Serovar 2 strain challenge. PLoS ONE.

[40] Li M., Wang C., Feng Y., Pan X., Cheng G., Wang J., Ge J., Zheng F., Cao M., Dong Y. (2008). SalK/SalR, a two-component signal transduction system, is essential for full virulence of highly invasive *Streptococcus suis* serotype 2. PLoS ONE.

[41] Tan M.F., Gao T., Liu W.Q., Zhang C.Y., Yang X., Zhu J.W., Teng M.Y., Li L., Zhou R. (2015). MsmK, an ATPase, contributes to utilization of multiple carbohydrates and host colonization of *Streptococcus suis*. PLoS ONE.

[42] Wang S., Yang Y., Zhao Y., Zhao H., Bai J., Chen J., Zhou Y., Wang C., Li Y. (2016). Sub-MIC tylosin inhibits *Streptococcus suis* biofilm formation and results in differential protein expression. Front. Microbiol..

[43] Laurenceau R., Krasteva P.V., Diallo A., Ouarti S., Duchateau M., Malosse C., Chamot-Rooke J., Fronzes R. (2015). Conserved *Streptococcus pneumoniae* spirosomes suggest a single type of transformation pilus in competence. PLoS Pathog..

[44] Yu Y., Qian Y., Du D., Xu C., Dai C., Li Q., Liu H., Shao J., Wu Z., Zhang W. (2016). SBP2 plays an important role in the virulence changes of different artificial mutants of *Streptococcus suis*. Mol. Biosyst..

[45] Fulde M., Willenborg J., de Greeff A., Benga L., Smith H.E., Valentin-Weigand P., Goethe R. (2011). ArgR is an essential local transcriptional regulator of the arcABC operon in *Streptococcus suis* and is crucial for biological fitness in an acidic environment. Microbiology.

[46] Haikarainen T., Thanassoulas A., Stavros P., Nounesis G., Haataja S., Papageorgiou A.C. (2011). Structural and thermodynamic characterization of metal ion binding in *Streptococcus suis* Dpr. J. Mol. Biol..

[47] Norris M.H., Rahman Khan M.S., Schweizer H.P., Tuanyok A. (2017). An avirulent *Burkholderia pseudomallei* purM strain with atypical type B LPS: Expansion of the toolkit for biosafe studies of melioidosis. BMC Microbiol..

[48] de Buhr N., Stehr M., Neumann A., Naim H.Y., Valentin-Weigand P., von Kockritz-Blickwede M., Baums C.G. (2015). Identification of a novel DNase of *Streptococcus suis* (EndAsuis) important for neutrophil extracellular trap degradation during exponential growth. Microbiology.

[49] Zheng C., Ren S., Xu J., Zhao X., Shi G., Wu J., Li J., Chen H., Bei W. (2017). Contribution of NADH oxidase to oxidative stress tolerance and virulence of *Streptococcus suis* serotype 2. Virulence.

