# Supplementary material for: Screening of Virulence-Related Transcriptional Regulators in Streptococcus suis
Source: Genes (Basel). 2020 Aug 21;11(9):972. doi: 10.3390/genes11090972 (PMC7564649; doi:10.3390/genes11090972)
Supplement: Supplementary file 1 [file genes-11-00972-s001.zip › Figure S5.pdf]

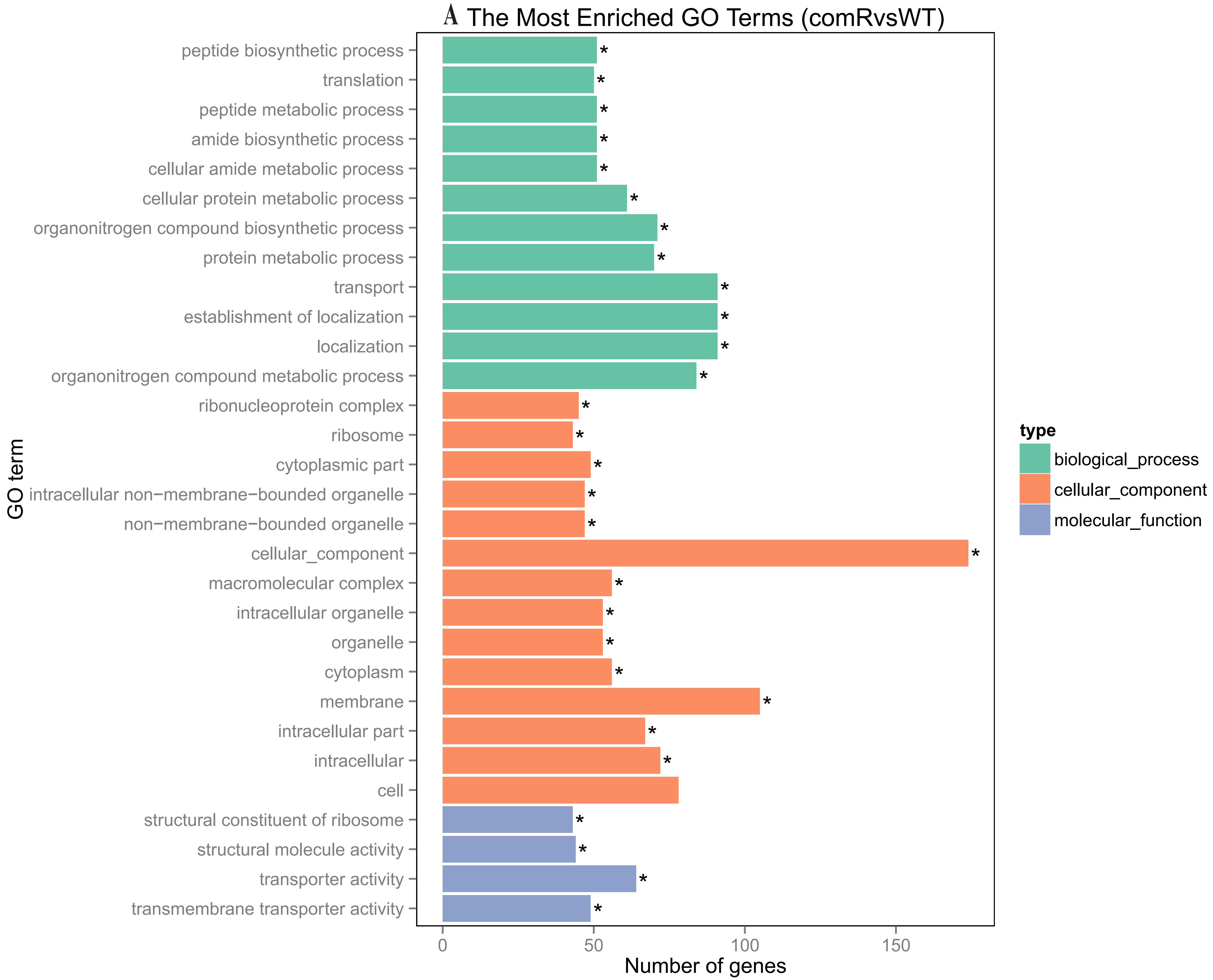

A The Most Enriched GO Terms (comRvsWT)
GO term
peptide biosynthetic process
translation
peptide metabolic process
amide biosynthetic process
cellular amide metabolic process
cellular protein metabolic process
organonitrogen compound biosynthetic process
protein metabolic process
transport
establishment of localization
localization
organonitrogen compound metabolic process
ribonucleoprotein complex
ribosome
cytoplasmic part
intracellular non−membrane−bounded organelle
non−membrane−bounded organelle
cellular_component
macromolecular complex
intracellular organelle
organelle
cytoplasm
membrane
intracellular part
intracellular
cell
structural constituent of ribosome
structural molecule activity
transporter activity
transmembrane transporter activity
0
50
100
150
Number of genes
type
biological_process
cellular_component
molecular_function

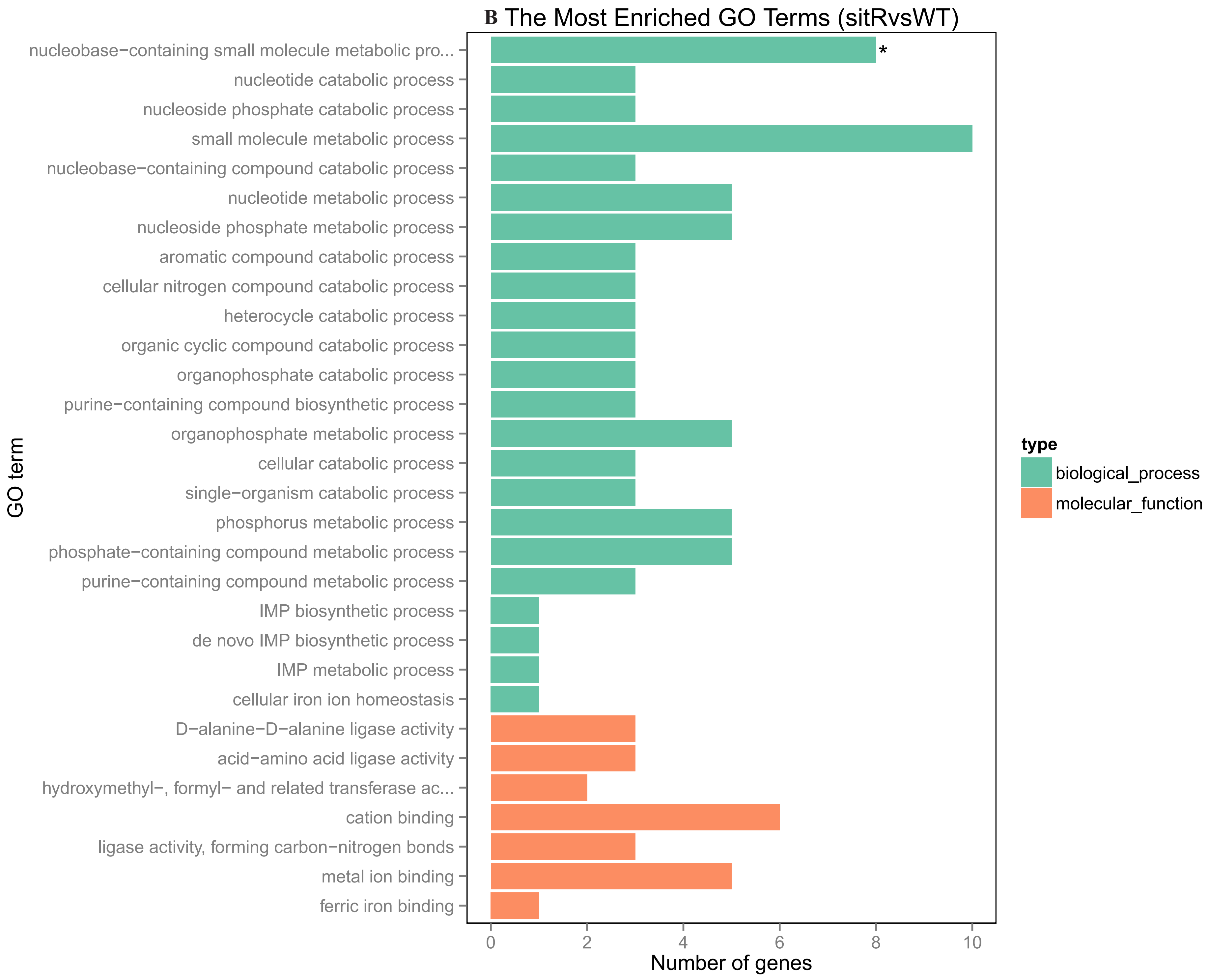
B The Most Enriched GO Terms (sitRvsWT)
nucleobase−containing small molecule metabolic pro...
*
nucleotide catabolic process
nucleoside phosphate catabolic process
small molecule metabolic process
nucleobase−containing compound catabolic process
nucleotide metabolic process
nucleoside phosphate metabolic process
aromatic compound catabolic process
cellular nitrogen compound catabolic process
heterocycle catabolic process
organic cyclic compound catabolic process
organophosphate catabolic process
purine−containing compound biosynthetic process
organophosphate metabolic process
cellular catabolic process
single−organism catabolic process
phosphorus metabolic process
phosphate−containing compound metabolic process
purine−containing compound metabolic process
IMP biosynthetic process
de novo IMP biosynthetic process
IMP metabolic process
cellular iron ion homeostasis
D−alanine−D−alanine ligase activity
acid−amino acid ligase activity
hydroxymethyl−, formyl− and related transferase ac...
cation binding
ligase activity, forming carbon−nitrogen bonds
metal ion binding
ferric iron binding
GO term
0
2
4
6
8
10
Number of genes
type
biological_process
molecular_function

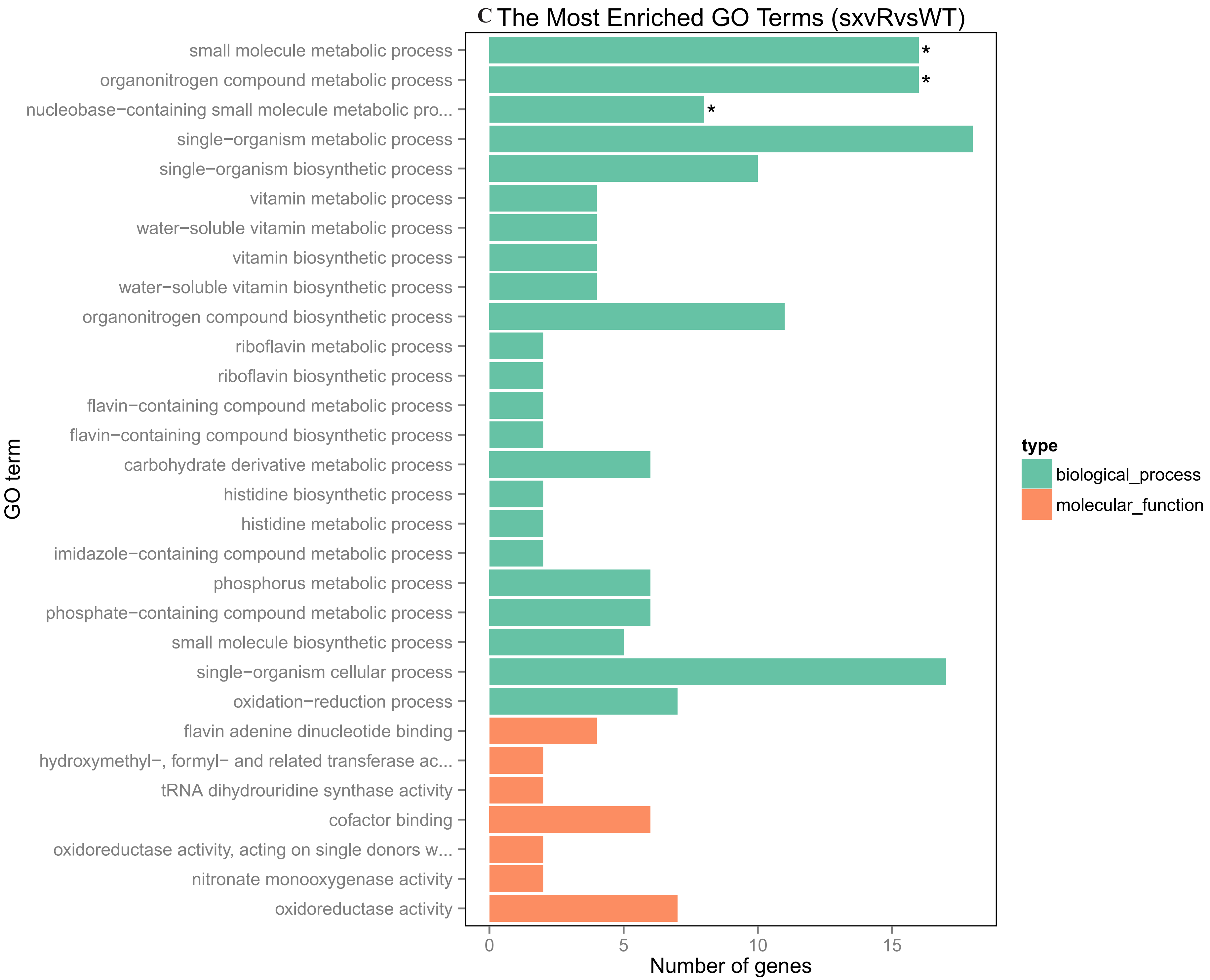
C The Most Enriched GO Terms (sxvRvsWT)
GO term
small molecule metabolic process
organonitrogen compound metabolic process
nucleobase−containing small molecule metabolic pro...
single−organism metabolic process
single−organism biosynthetic process
vitamin metabolic process
water−soluble vitamin metabolic process
vitamin biosynthetic process
water−soluble vitamin biosynthetic process
organonitrogen compound biosynthetic process
riboflavin metabolic process
riboflavin biosynthetic process
flavin−containing compound metabolic process
flavin−containing compound biosynthetic process
carbohydrate derivative metabolic process
histidine biosynthetic process
histidine metabolic process
imidazole−containing compound metabolic process
phosphorus metabolic process
phosphate−containing compound metabolic process
small molecule biosynthetic process
single−organism cellular process
oxidation−reduction process
flavin adenine dinucleotide binding
hydroxymethyl−, formyl− and related transferase ac...
tRNA dihydrouridine synthase activity
cofactor binding
oxidoreductase activity, acting on single donors w...
nitronate monooxygenase activity
oxidoreductase activity
*
*
*
0
5
10
15
Number of genes
type
biological_process
molecular_function
