# Supplementary material for: Screening of Virulence-Related Transcriptional Regulators in Streptococcus suis
Source: Genes (Basel). 2020 Aug 21;11(9):972. doi: 10.3390/genes11090972 (PMC7564649; doi:10.3390/genes11090972)
Supplement: Supplementary file 1 [file genes-11-00972-s001.zip › Table S1.docx]

**Table S1** Summary of bacterial strains and plasmid used in this study.

| **Group** | **Name** | **Characteristics**  **and functions** | **Sources or**  **references** |
| --- | --- | --- | --- |
| Bacterial strains |  |  |  |
|  | SC-19 | *Streptococcus suis* serotype 2 wild type | Laboratory collection |
|  | Δ0275 | SSUSC84_0275 deletion mutant strain | This study |
|  | Δ0315 | SSUSC84_0315 deletion mutant strain | This study |
|  | Δ0046 | SSUSC84_0046 deletion mutant strain | This study |
|  | Δ0874 | SSUSC84_0874 deletion mutant strain | This study |
|  | Δ1645 | SSUSC84_1645 deletion mutant strain | This study |
|  | Δ1787 | SSUSC84_1787 deletion mutant strain | This study |
|  | Δ0005 | SSUSC84_0005 deletion mutant strain | This study |
|  | Δ0111 | SSUSC84_0111 deletion mutant strain | This study |
|  | Δ0756 | SSUSC84_0756 deletion mutant strain | This study |
|  | CΔcomR | complemented strain of *hp0046* | This study |
|  | CΔsitR | complemented strain of *hp0874* | This study |
|  | CΔsxvR | complemented strain of *hp1787* | This study |
|  | *Escherichia coli* DH5α |  | TIANGEN |
| Plasmids |  |  |  |
|  | pSET4s | *E. coli–S. suis* shuttle vector; Spcr | Laboratory collection |
|  | pSET2 | *E. coli–S. suis* shuttle vector; Spcr | Laboratory collection |
