# Supplementary material for: Screening of Virulence-Related Transcriptional Regulators in Streptococcus suis
Source: Genes (Basel). 2020 Aug 21;11(9):972. doi: 10.3390/genes11090972 (PMC7564649; doi:10.3390/genes11090972)
Supplement: Supplementary file 1 [file genes-11-00972-s001.zip › Table S3.docx]

**Table S3** | KEGG analyses of the pathways involved in the DEGs of ΔcomR, ΔsitR and ΔsxvR

|  | Pathway | Input number | Background number | P-Value | Up/down |
| --- | --- | --- | --- | --- | --- |
| **ΔcomR vs WT** | | | | | |
|  | Ribosome | 49 | 68 | 1.721E-08 | down |
|  | ABC transporters | 40 | 75 | 8.7E-05 | down |
|  | 2-Oxocarboxylic acid metabolism | 10 | 15 | 0.0161661 | down |
|  | Fatty acid metabolism | 8 | 12 | 0.0305809 | down |
|  | Fatty acid biosynthesis | 8 | 13 | 0.041084 | down |
|  | Pyrimidine metabolism | 16 | 46 | 0.0061436 | up |
|  | Alanine, aspartate and glutamate metabolism | 8 | 15 | 0.0077337 | up |
|  | Metabolic pathways | 64 | 326 | 0.0373022 | up |
|  | Biosynthesis of secondary metabolites | 33 | 152 | 0.0472178 | up |
|  | Streptomycin biosynthesis | 4 | 7 | 0.0492072 | up |
| **ΔsitR vs WT** | | | | | |
|  | Purine metabolism | 9 | 51 | 5.333E-06 | down |
|  | Biosynthesis of secondary metabolites | 9 | 152 | 0.0100812 | down |
|  | Pyrimidine metabolism | 4 | 46 | 0.0275231 | down |
|  | One carbon pool by folate | 2 | 10 | 0.0305376 | down |
| **ΔsxvR vs WT** | | | | | |
|  | Pyrimidine metabolism | 9 | 46 | 0.0003067 | down |
|  | Purine metabolism | 8 | 51 | 0.0024285 | down |
|  | Alanine, aspartate and glutamate metabolism | 4 | 15 | 0.0066243 | down |
