# Supplementary figures and images for: Screening of Virulence-Related Transcriptional Regulators in Streptococcus suis
Source: Genes (Basel). 2020 Aug 21;11(9):972. doi: 10.3390/genes11090972 (PMC7564649; doi:10.3390/genes11090972)

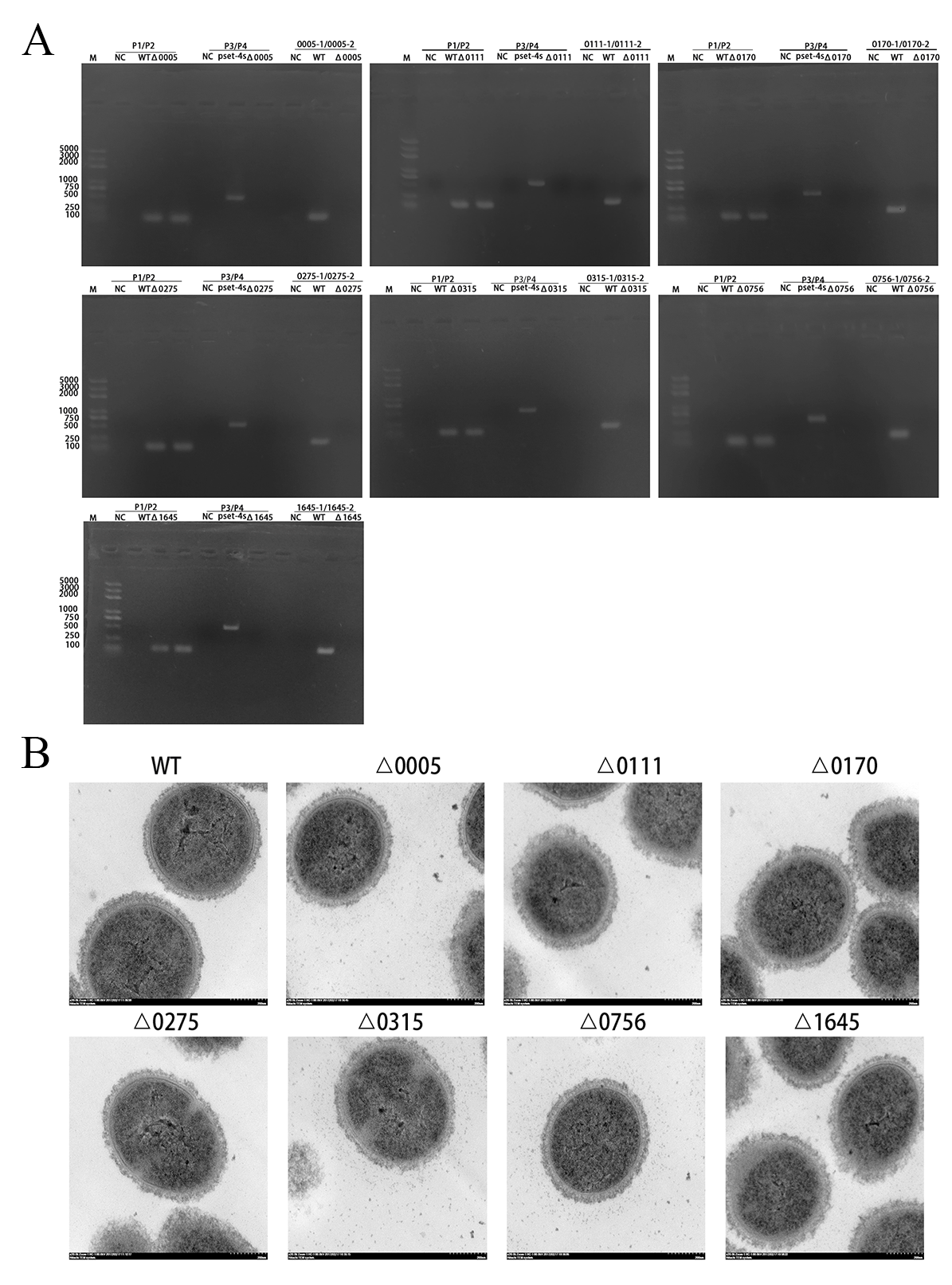

Supplement: Supplementary file 1 [file genes-11-00972-s001.zip › Figure S1.tif]

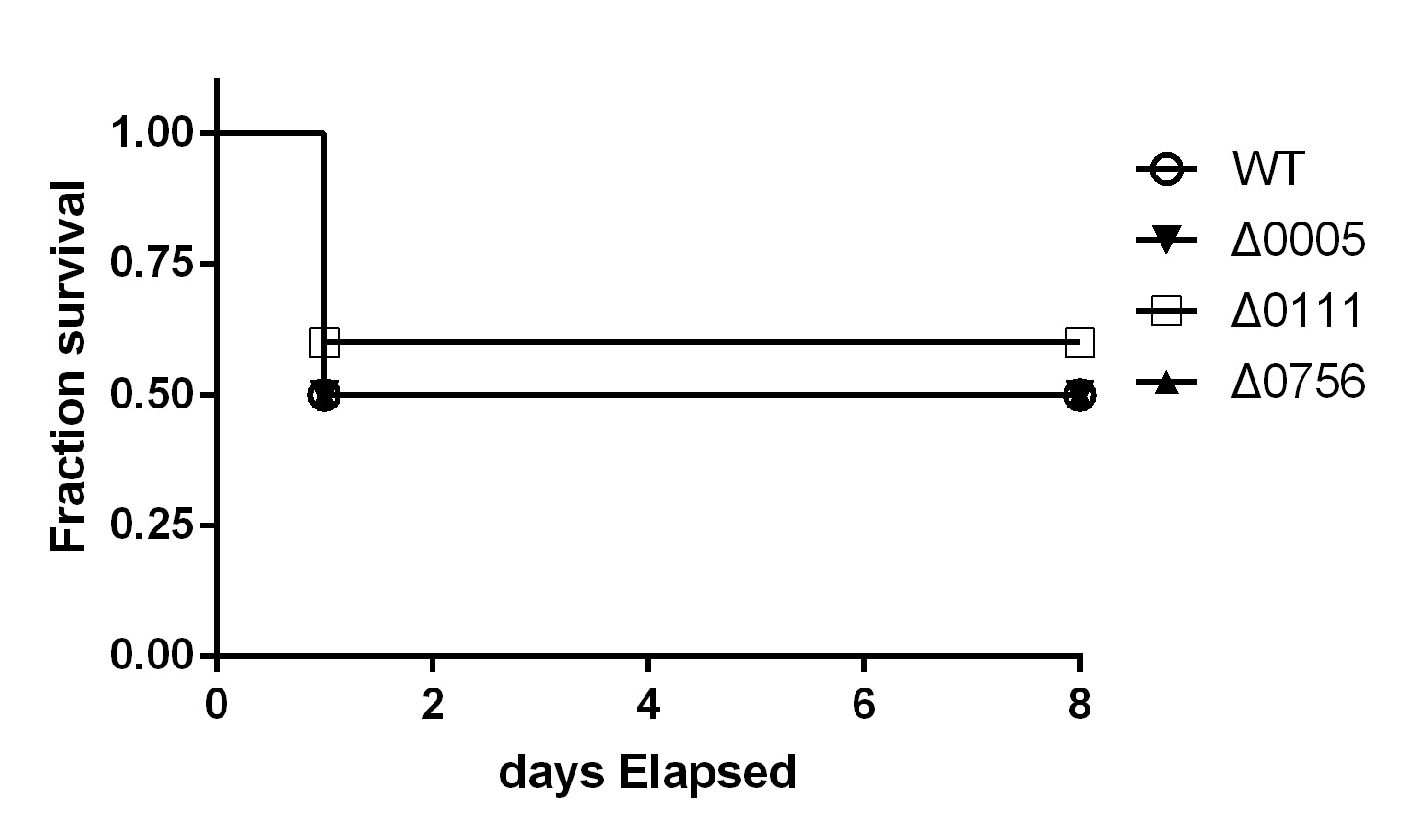

Supplement: Supplementary file 1 [file genes-11-00972-s001.zip › Figure S2.jpg]

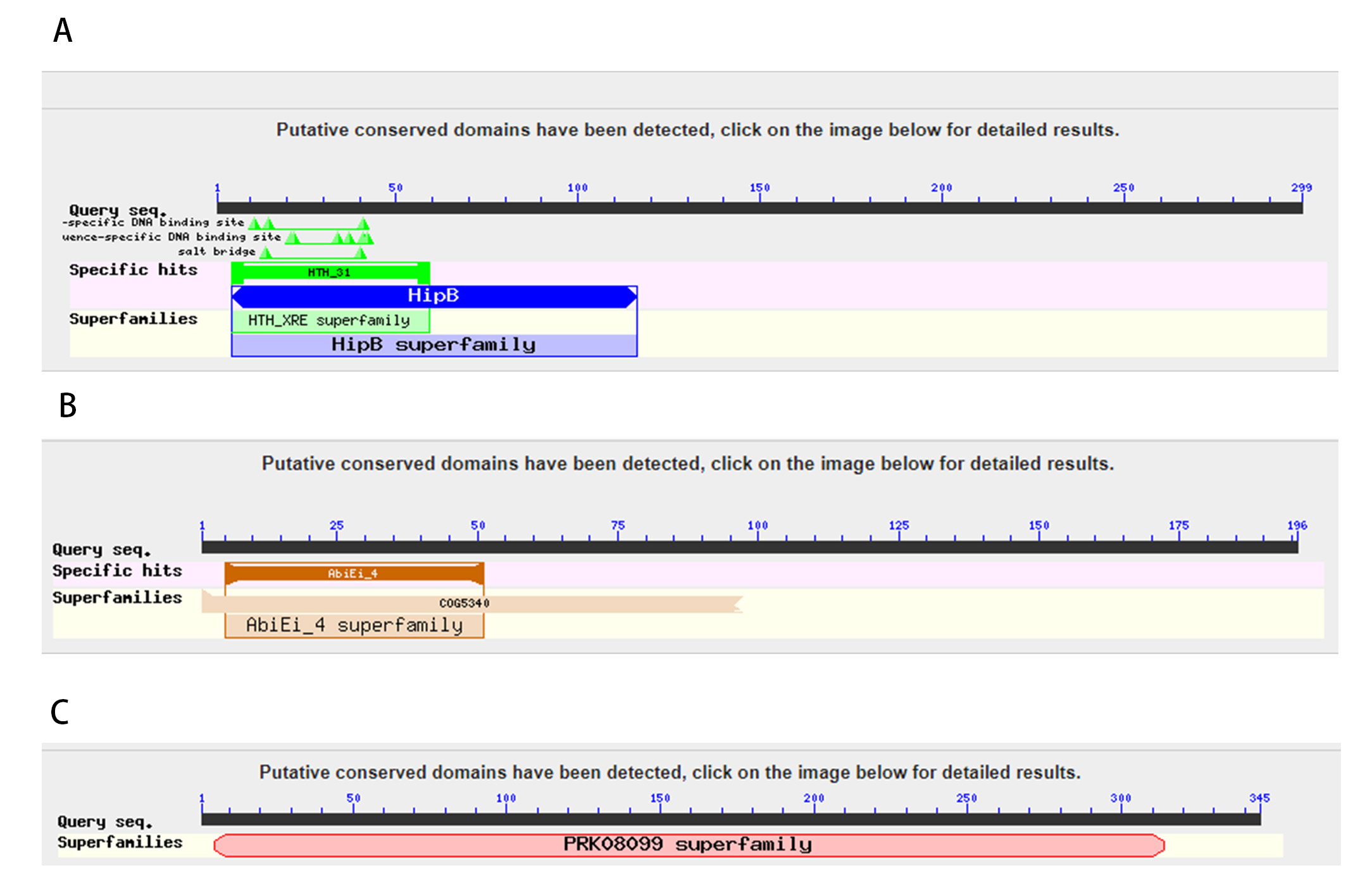

Supplement: Supplementary file 1 [file genes-11-00972-s001.zip › Figure S3.jpg]

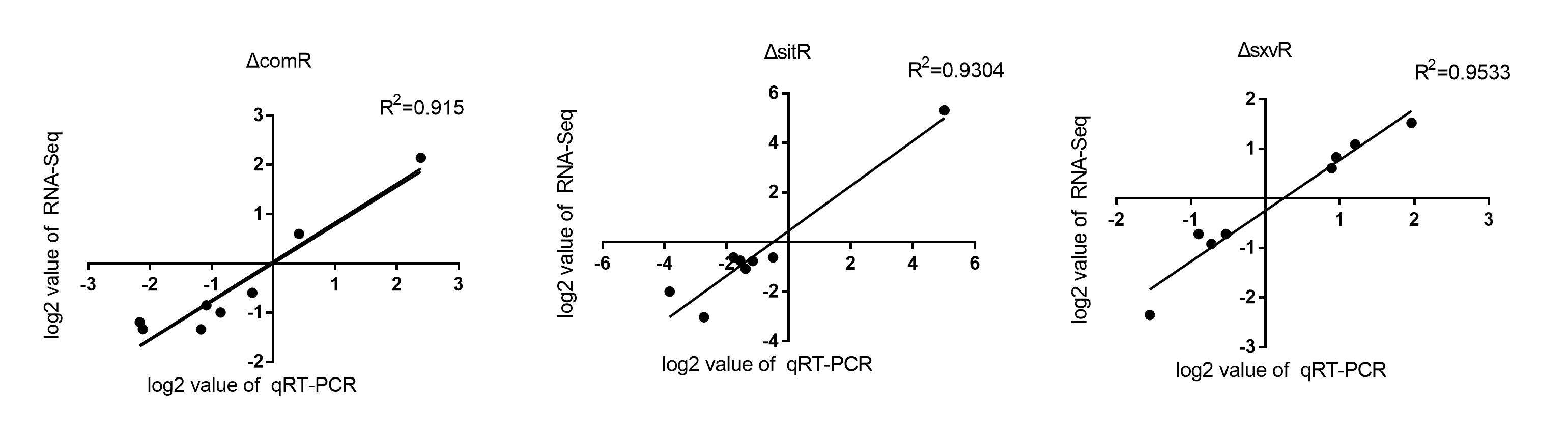

Supplement: Supplementary file 1 [file genes-11-00972-s001.zip › Figure S4.jpg]
